# Update on the Role of FeNO in Asthma Management

**DOI:** 10.3390/diagnostics13081428

**Published:** 2023-04-15

**Authors:** Neveda Murugesan, Damini Saxena, Arundhati Dileep, Muhammad Adrish, Nicola A. Hanania

**Affiliations:** 1 Section of Pulmonary, Critical Care and Sleep Medicine, Baylor College of Medicine, Houston, TX 77030, USA; 2 Division of Pulmonary & Critical Care Medicine, Bronx Care Health System, Bronx, NY 10457, USA

**Keywords:** fractional excretion of nitric oxide, FeNO, asthma, outcomes

## Abstract

Asthma is a heterogenous disorder characterized by presence of different phenotypes and endotypes. Up to 10% of the individuals suffer from severe asthma and are at increased risk of morbidity and mortality. Fractional exhaled nitric oxide (FeNO) is a cost-effective, point of care biomarker that is used to detect type 2 airway inflammation. Guidelines have proposed to measure FeNO as an adjunct to diagnostic evaluation in individuals with suspected asthma and to monitor airway inflammation. FeNO has lower sensitivity, suggesting that it may not be a good biomarker to rule out asthma. FeNO may also be used to predict response to inhaled corticosteroids, predict adherence and deciding on biologic therapy. Higher levels of FeNO have been associated with lower lung function and increased risk for future asthma exacerbations and its predictive value increases when combined with other standard measurements of asthma assessment.

## 1. Introduction

Asthma is a heterogenous chronic respiratory condition that impacts all ethnicities and age groups. It also poses a significant global health burden affecting approximately 358 million people worldwide [1]. Asthma leads to significant morbidity and is the 2nd leading cause of death among the chronic respiratory condition [2]. The diagnosis can be challenging as symptoms are non-specific and demonstration of variable airflow limitation can be difficult to obtain, particularly among the children. In individuals with mild asthma, airflow obstruction may not be seen during spirometry and may require other modalities such as serial peak flow measurements or bronchial provocation testing. Peak flow testing can have limited diagnostic value whereas bronchial provocation testing is time-intensive, costly and can only be performed in specialized laboratories [3]. 

Type 2 inflammation is prevalent in asthma and can be found in up to 80% of corticosteroid-naïve individuals [4]. Sputum eosinophilia is a marker of eosinophilic airway inflammation and has been shown to predict asthma outcomes [5]. However, the test is also cumbersome due to the requirement of laboratory facilities and the time it takes to perform the test. Fractional exhaled nitric oxide (FeNO) measurement and blood eosinophil count (BEC) have been considered as surrogate markers for assessing eosinophilic airway inflammation. BEC, which is an invasive test, has been shown to have moderate-to-good correlation with sputum eosinophilia in individuals with asthma [6]. On the other hand, FeNO has a modest correlation with sputum eosinophilia especially in individuals who have steroid-dependent asthma [7,8]. 

FeNO presents several advantages compared to other diagnostic and management modalities for asthma. It is a non-invasive test that can be done quickly [9]. The test is simple to perform and is well tolerated by adults and pediatric individuals with asthma. FeNO has also been shown to have moderate diagnostic accuracy for asthma and can be used to monitor disease activity. FeNO can be used to select treatment agents, monitor response to treatment and assist with change in therapy. In this review we will discuss the role of nitric oxide in type-2 (T2) inflammation and clinical utility of FeNO testing in individuals with asthma. 

## 2. Role of Nitric Oxide in Type 2 Inflammation 

Environmental nitric oxide (NO) is a colorless, highly reactive gas found in the environment, particularly in motor vehicle exhaust and cigarette smoke [10]. However, nitric oxide is also ubiquitous in all human organ systems and has myriad functions: a vasodilator, a bronchodilator, a neurotransmitter, and an inflammatory mediator. Its free radical properties make it highly bactericidal and cytotoxic. Interestingly, in the respiratory system, NO acts as both proinflammatory and anti-inflammatory [10]. This duality of NO is key to understanding its role in T2 airway inflammation in asthma.

In the lungs, NO is produced by epithelial cells, vascular endothelial cells, and neurons [11]. Macrophages and neutrophils are also capable of producing NO [4]. NO can be produced enzymatically and non-enzymatically [11,12,13]. The mechanism of non-enzymatic production of NO in the airways is less understood but incorporates reduction in nitrate to nitrite, which converts to NO [12]. This process occurs more readily in acidic environments; nitrite combines with hydrogen ions to form nitrous oxide that disintegrates into NO. NO is also formed enzymatically in the airways from L-arginine, which is taken up by airway epithelial cells and is converted to NO by NO synthase (NOS) [11,12,13]. There are three isoforms of NOS in the lungs: constitutive neuronal NOS (nNOS), constitutive endothelial NOS (eNOS), and inducible NOS (iNOS). The constitutive form of NOS is aptly known as cNOS and produces low amounts of NO in short bursts. The expression of iNOS is transcription-dependent and, in contrast, produces large amounts of NO for prolonged periods.

Physiologic production of NO by cNOS leads to smooth muscle relaxation, while inhibiting smooth muscle proliferation and inflammation [13]. The former is accomplished through the indirect production of cGMP by NO-activated guanylyl cyclase and subsequent activation of protein kinases [12,14]. The overall effect is airway bronchodilation. NO can also interact directly with high energy free radicals, mitigating oxidative stress and generation of proinflammatory lipids, thereby reducing inflammation [12]. Production of NO by eNOS helps enhance lung development, promotes ciliary motility, produces surfactant, and protects against bronchoconstriction [11,14]. Physiologic production of NO by iNOS assists in innate immunity against external pathogens and malignant cells due to NO’s cytotoxicity at high concentrations [11].

In T2 asthma, production of NO is altered, leading to airway hyperresponsiveness. Animal studies have shown that after allergen exposure in asthmatic airways, there is a deficiency in NO production by cNOS, resulting in bronchoconstriction [13]. This deficiency may be in part due to reduced uptake of L-arginine by epithelial cells and hence reduced substrate availability for cNOS [13]. Furthermore, NOS enzymes coexist in competition with other enzymes that reduce available substrate for NO production, such as arginase, which converts L-arginine into L-ornithine [5]. T-helper 2 (Th2) cytokines increase expression of arginase, causing even more loss of substrate available for NOS to produce NO. NOS production of NO is further reduced in the presence of asymmetric dimethyl arginine (ADMA), an analogue of L-arginine, which competitively inhibits iNOS [2]. Some studies also suggest that cNOS expression is downregulated, contributing to airway hyperresponsiveness [13].

Production of the cNOS-derived NO is reduced, while iNOS induced NO levels increase that cause paradoxical bronchoconstriction and airway inflammation. Furthermore, the presence of proinflammatory cytokines, such as tumor necrosis factor, interferon-gamma, and interleukin (IL)-1β, due to allergen exposure triggers overexpression of iNOS [12,13]. Excessive production of NO amplifies its physiologic functions markedly, leading to hyperemia, hypotension, and edema. NO derived from iNOS perpetuates T2 inflammation, causing lung epithelial damage, mucous hypersecretion, vascular permeability, and eosinophilia [12].

NO produced by iNOS results in “nitrative stress” that adds to the type 2 inflammation seen in individuals with asthma. Under oxidative stress of an inflammatory state, NO reacts with superoxide anions to form reactive nitrogen oxide species (RNOS), including peroxynitrite and dinitrogen trioxide [11]. ADMA’s inhibitory effect of iNOS contributes to oxidative stress with the generation of oxygen oxide and downstream RNOS [2]. RNOS inflict airway cellular damage, cause DNA, protein, and mitochondrial dysfunction, and promote airway hyperresponsiveness in individuals with asthma [13].

Therefore, appropriate levels of NO are integral in regulating lung mechanics and airway inflammation. At low constitutive levels, NO helps promote bronchodilation and reduces inflammation. In type 2 inflammation in individuals with asthma, NO is produced in pathologic amounts by iNOS. Under oxidative stress, its protective effect disappears, resulting in bronchial hyperreactivity, mucus hypersecretion, vasodilation, increased vascular permeability and various proinflammatory effects including free radical production leading to cytotoxicity [15]. Elevated FeNO levels have been seen in acute and chronic airway inflammation and reflects IL-4 and IL-13 induced induction of iNOS. Increased iNOS activity favors Th2 lymphocyte differentiation and inhibits Th1 and Th17 lymphocytes as well as IL-12 synthesis [15]. 

## 3. FeNO as a Diagnostic Biomarker for Asthma 

To date, asthma remains a clinical diagnosis and there is no single diagnostic test for the disease. Confirming the diagnosis requires a high degree of clinical suspicion and can be challenging given its various phenotypes. International guidelines recommend the use of bronchoprovocation testing if lung function is normal to further evaluate symptoms suggestive of asthma, but this can labor-intensive, time-consuming, expensive and comes with the risk of acute bronchospasm for individuals [16,17].

FeNO is an established biomarker that reflects underlying respiratory tract inflammation and provides an easy, non-invasive and reproducible way to detect airway inflammation in some individuals with asthma [11]. Studies have shown that even in milder stages of asthma, individuals can have higher FeNO concentrations than normal individuals in their exhaled air [18,19]. Despite this, FeNO has not been adopted as a standard tool for the diagnosis of asthma. A meta-analysis by Karrasch and colleagues investigated this issue and concluded that the specificity of FeNO levels were higher than its sensitivity, suggesting that a FeNO measurement would be more accurate in ruling in rather than ruling out the diagnosis of asthma [20]. Other studies have also evaluated the role of FeNO in predicting asthma diagnosis in combination with other measurements. A recent study by Louis and colleagues demonstrated that a combination of wheezing scale, spirometry and FeNO improves the diagnostic accuracy with area under curve of 0.76 (95% confidence interval 0.66–0.84) [21]. In another study of 58 adults with persistent allergic rhinitis followed over 1 year showed that basal FeNO levels over 28 parts per billion (ppb) predicted development of asthma [22]. Similar findings were also noted in children where FeNO was noted to be helpful in identifying wheezing phenotypes in preschool children. Children with increased FeNO levels during preschool years had increased risk of asthma and impaired lung growth later in life [23]. 

However, use of FeNO remains an adjuvant for diagnosis because several other disorders can affect FeNO levels. Conditions such as chronic rhinosinusitis (CRS), allergic rhinitis are similarly characterized by eosinophilic inflammation, leading to increased FeNO values [12]. A cross-sectional study by Duarte and colleagues noted that FeNO measurements in obstructive sleep apnea (OSA) were also elevated, possibly correlating with increased upper airway inflammation found in the disease [24]. Furthermore, non-disease related factors, such as older age, height, male sex, ethnicity, smoking history, viral infections, certain nitrate rich foods (especially leafy vegetables), and use of anti-inflammatory medications, have all been correlated with higher FeNO levels [11]. 

**Figure 1:**
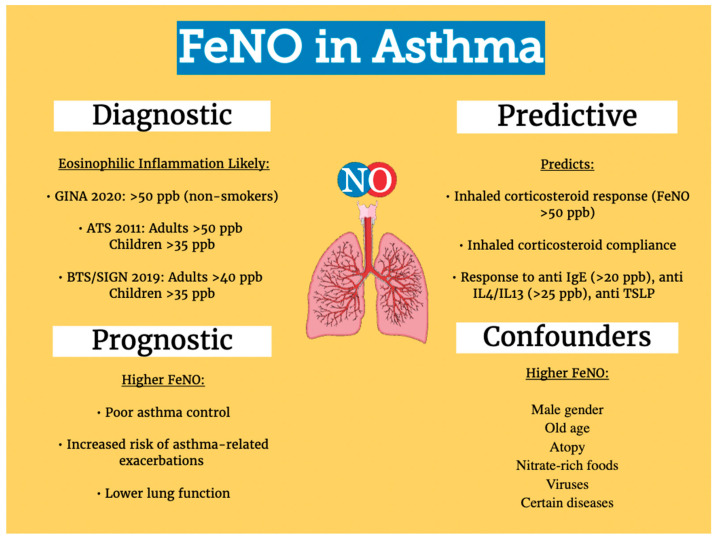
Diagnostic, prognostic, predictive value, and common confounders of FeNO use in asthma.

Consequently, the interpretation of FeNO levels is highly dependent on an individual’s clinical history and symptoms. The official practice guidelines published by the American Thoracic Society (ATS) in 2011 considers these multiple confounding factors and suggests the use of cutoff points rather than set reference ranges. Since FeNO tends to be higher in healthy adults than children (defined as less than twelve years of age), ATS proposes two sets of cutoff points for adults and children [10,25]. In symptomatic adults and children with FeNO concentrations below 25 ppb and 20 ppb, respectively, eosinophilic inflammation is less likely, and alternative diagnoses such as chronic cough, gastroesophageal reflux disease (GERD), vocal cord dysfunction or even non-allergic asthma should be considered. Conversely, FeNO levels above 50 ppb in adults and levels above 35 ppb in children with symptoms are thought to have significant eosinophilic inflammation and supports a diagnosis of asthma (Figure 1). Of note, there is an intermediate range of FeNO concentrations, from 25 to 50 ppb in adults and from 20 to 35 ppb in children, which should be interpreted within the clinical context, and indeed tracking FeNO concentration levels over time may reveal an asthma diagnosis rather than one isolated positive test [10]. 

However, it is important to note that these cut-points vary between international guidelines. Current National Institute of Health and Clinical Excellence (NICE) guidelines in the United Kingdom (UK) recommend using FeNO levels above 40 ppb and 35 ppb in adults and children between 5–16 years of age, respectively. According to The Global initiative for Asthma (GINA) strategy, a FeNO concentration equal to or above 20 ppb is considered high alongside other biomarkers indicative of T2 immune response, like BEC ≥150 cells/μL and or sputum eosinophils ≥2% [12]. The differences in cutoff values are likely explained by the data used by each group, but again highlight how FeNO levels can be influenced by many etiologies other than asthma. 

In their recent report, the National Asthma Education and Prevention Program Expert Panel Report 4 (EPR-4) recommended using FeNO as an adjunct to the diagnostic evaluation of individuals where asthma diagnosis is uncertain [26]. A FeNO cutoff value of >50 ppb was proposed for non-smoking adults and a cutoff of >35 ppb was proposed for children 5–12 years of age who were not using corticosteroids. The 2022 guidelines for diagnosis of asthma by European Respiratory Society (ERS) also gave a conditional recommendation for using FeNO is the diagnostic workup with a suggested cutoff of 40 ppb due to its best compromise between sensitive and specificity [27]. GINA also suggests that a FeNO level above 20 ppb can diagnose type 2 inflammation in individuals who have difficult-to-treat or severe asthma [28].

Individuals with asthma can also have other comorbidities that can affect FeNO levels. Chronic rhinosinusitis and nasal polyposis are common in individuals with asthma and FeNO can predict nasal polyposis even in the absence of blood eosinophilia. Similarly, FeNO levels can also be elevated in the presence of allergic rhinitis and nasal steroids can decrease FeNO. FeNO levels are low in the presence of bronchiectasis and gastroesophageal reflux disease, whereas the levels are not affected by obesity and obstructive sleep apnea syndrome [29]. 

In summary, studies demonstrate that FeNO levels are subject to many confounding factors and although there are suggested cutoff points proposed by various guidelines, interpretation and application of the test should always be used in the clinical context of the patient’s history and symptoms. Further efforts to determine subject-specific FeNO cut-points are in progress, and hopefully evolving research will provide further diagnostic accuracy of FeNO for asthma. 

## 4. FeNO as a Biomarker of Predicting Asthma Control, Exacerbation and Lung Function Decline 

Several studies demonstrated the ability of FeNO in predicting airway inflammation and risk of exacerbation and poor asthma control. In a study by Pavlidis and colleagues, a FeNO levels of ≥30 ppb gave a moderate prediction of T2 high asthma [30]. Elevated FeNO levels have also been associated with asthma control and risk of asthma exacerbation [31]. In a German cohort of individuals with severe asthma where elevated FeNO levels were associated with poor asthma control, lower lung function and increased frequency of asthma exacerbations [32]. In this study, FeNO levels of ≥25 ppb had 65% sensitivity in predicting two or more annual exacerbations. Similarly, in a British study of 115 individuals with severe asthma, FeNO levels demonstrated a stronger correlation with acute exacerbation compared BEC or serum periostin levels [33]. 

Studies have shown conflicting results by failing to demonstrate any association between FeNO and asthma control. In a study of 82 individuals with asthma, low FeNO levels were associated with uncontrolled asthma [34]. Another study from Japan evaluated role of FeNO during the treatment period in individuals with asthma and failed to demonstrate any association between FeNO and deterioration in asthma [35]. Similar findings were noted in several other studies [36,37,38]. A metanalysis which included 175 studies, showed a weak association between FeNO and asthma control [39]. 

These differences between association of FeNO and asthma control can be explained by reviewing the clinical characteristics of individuals included in the different studies [12]. Individuals who were not on maintenance treatment demonstrated better correlation between elevated FeNO levels and poor asthma control [12]. Conversely, individuals with asthma who were on maintenance treatment had weaker or no correlation between asthma control and FeNO [36,38]. In a review by Ulrik and colleagues that included 35 studies, high FeNO levels were associated with accelerated lung function decline in adults with long standing moderate to severe asthma regardless of optimal treatment. These findings were less robust in individuals with less severe disease. Nonetheless, the authors noted that FeNO based management may decrease risk of asthma-related exacerbations in adults [18]. 

The relationship of BEC and FeNO as a prognostic biomarker and in predicting risk of asthma exacerbation has been another area of interest. In an observational study by Price and colleagues, individuals with asthma who had elevated FeNO (≥50 ppb) or elevated BEC (≥300 cells/μL) had higher risk of exacerbation (risk ratio 1.31). When both biomarkers were combined, the risk ratio for acute exacerbation increased to 3.67 [40]. Similar findings of combination of FeNO and BEC in predicting asthma exacerbation were also noted in a post-hoc analysis of data from Liberty Quest study [41]. 

The role of FeNO alongside lung function parameters in predicting asthma control has also been explored. In a study of 662 children, spirometry adjusted FeNO/FEV1 demonstrated an increased ability to identify uncontrolled asthma compared to FeNO alone (area under curve = 0.707; *p* = 0.011) [42]. A study from Korea combined FeNO ≥35 ppb and bronchodilator response and demonstrated that the combination performed better than individual variables in predicting loss of asthma control [43]. Higher FeNO levels have also been associated with lung function decline. In a study of adults with newly diagnosed asthma a FeNO cutoff of ≥57 ppb was associated with more rapid lung function decline [44]. Similar findings were also noted in Japanese and Korean cohorts [45,46]. Combining elevated FeNO levels and BEC had higher odds ratio for predicting lower lung function than either of the measurement alone [47]. Together these findings suggest that FeNO may have better predictive value in assessing asthma control and progression when combined with other measurements. 

## 5. FeNO as a Biomarker to Guide Inhaled Corticosteroids Therapy 

Inhaled corticosteroids (ICS), which are the mainstay of asthma treatment, act by reducing T2 airway inflammation. Use of FeNO has been shown to predict response to ICS therapy, however, cutoff points for adjusting treatment are not well established. In a double-blind, randomized, controlled trial of undiagnosed adults with symptoms suggestive of asthma, individuals were assigned to treatment with inhaled corticosteroid or placebo [48]. The randomization was stratified by FeNO cutoffs including ≤25 ppb, >25 to <40 ppb and ≥40 ppb. Asthma Control Questionnaire (ACQ7) score was the primary endpoint. A significant interaction was observed between baseline FeNO and treatment arms for every 10-ppb change in FeNO. The effect was more pronounced in the ICS group compared to placebo group. A retrospective study evaluated the relationship between response to ICS and FeNO measurements and found a cutoff of 38 ppb was able to differentiate between ICS responders and non-responders [49]. Another observational study compared treatment decision based on physician’s assessment of patient’s symptoms, physical examination and spirometry and found that without FeNO measurements, airway inflammation was incorrectly assessed in half of the individuals. Adding FeNO to clinical assessment modified treatment decisions in 36% of the individuals in this study [50]. ATS guidelines recommend that FeNO levels of >50 ppb in adults can be used to indicate responsiveness to corticosteroids [10]. 

The FeNO-based management of asthma has also been evaluated during pregnancy. A study by Tamasi and colleagues evaluated use and reproducibility of FeNO in 102 females which included 20 pregnant asthmatic women, 20 nonpregnant asthmatic women, 35 healthy nonpregnant women and 27 healthy pregnant women [51]. The authors concluded that the FeNO levels were not influenced by pregnancy. In a double blind, parallel group, controlled trial, 220 pregnant individuals with asthma were randomly assigned before 22 weeks gestation to a treatment guided by clinical symptoms or FeNO levels. FeNO level of >29 ppb and <16 ppb was used to up titrate and down titrate ICS dose, respectively. The exacerbation rate was lower in the FeNO group with a number needed to treat was 6. The FeNO group also had improvement in quality of life and reduced neonatal hospitalizations [52]. In another double-blind randomized controlled trial, FeNO guided management during pregnancy decreased incidence of doctor diagnosed asthma in the offspring at pre-school age [53]. This was in part due to modification in the use of ICS during the trial period.

Suppression of FeNO after treatment with ICS can be used to monitor therapy and establish adherence [54]. McNicholl and colleagues used FeNO to identify nonadherence by using directly observed inhaled corticosteroid treatment (DOICS) [55]. In their study, individuals with asthma who had FeNO >45 ppb were divided into adherent (filling ICS prescription >80%) and nonadherent group (filling ICS prescription <50%). They received seven days of DOICS therapy with budesonide and were tested for nonadherence based on changes in FeNO. Nonadherent individuals had significantly greater drop in FeNO levels compared to adherent individuals. In another study that used FeNO suppression test via remote monitoring technology, demonstrated that it was useful in assessing adherence to the treatment [56]. It is also pertinent to mention here that up to 1/3rd of individuals with asthma can have elevated FeNO despite adequate corticosteroid therapy [54]. 

Finally, FeNO measurements may also facilitate stepdown from ICS therapy in individuals whose asthma symptoms are well controlled. In a recent metanalysis representing 384 participants from seven studies, FeNO level of <50 ppb was an appropriate cutoff for ICS dose reduction without increasing risk for exacerbations [57]. Studies have explored utility and cost effectiveness of monitoring FeNO levels in individuals with asthma due to its ability to improve diagnostic accuracy, monitor adherence and response to treatment [58]. A Swedish study evaluated the economic impact of FeNO use to diagnose and manage asthma in primary care setting and found that its addition led to cost saving of 672 Swedish Krona per patient by the 4th year. These findings suggest that FeNO use in managing asthma in primary care setting is feasible and cost-effective compared to other standard asthma management methods [59]. 

## 6. FeNO as a Predictive and Pharmacodynamic Biomarker in Targeted Biologic Therapy 

T2 high asthma is primarily driven by cytokines (IL-4, IL-5, IL-13), eosinophil alarmins (IL-25, IL-33, thymic stromal lymphopoietin [TSLP]), and Immunoglobulin E (IgE) [60]. FeNO is a noninvasive biomarker in individuals with T2 airway inflammation [15,61].

Currently, six Food and Drug Administration (FDA) approved biologic agents are available for treating individuals with severe asthma. Due to lack of high-level evidence from randomized trial comparing the efficacy of biologic agents with each other, decision to choose initial biologic agent or which agent to select when initial therapy fails often relies on several pieces of information including baseline biomarker levels [62]. These biomarkers including FeNO, sputum eosinophils, BEC, and IgE are often used when encountered with these decisions.

Omalizumab was one of the first biologic therapies approved for asthma management. It is an anti-IgE recombinant monoclonal antibody that binds to the free IgE, which is released in the presence of environmental allergens. It is approved to treat adults and children six years and older with moderate or severe persistent asthma who are not well controlled on standard treatment with ICS. In the U.S., it is approved in individuals with a total IgE level between 30 to 700 IU/mL in adults and 30–1300 IU/mL in children 6–11 years of age. Additionally, evidence of in vitro perennial aeroallergen reactivity should be noted, or a positive skin test to qualify with a lack of other underlying conditions that could lead to IgE elevation [63].

In A Study of Omalizumab (Xolair) in Subjects with Moderate to Severe Persistent Asthma (EXTRA), individuals with a pretreatment FeNO ≥24 ppb had a more favorable response to the treatment with omalizumab [64]. The investigators noted that reduction in exacerbation rates was higher in individuals with high versus low FeNO (53% versus 16%, respectively). Additionally, an increase in FeNO at week 12 after treatment interruption could predict future exacerbation rates. In this set of individuals resuming treatment with omalizumab could be beneficial to reduce the risk of exacerbations. Even when several other serum biomarkers (specific-to-total IgE ratios, serum tryptase, eosinophil cationic protein, or soluble CD23) were compared, they were unable to predict response to omalizumab [65]. However, in a prospective real-world study with omalizumab; Prospective Observational Study to evaluate Predictors of clinical Effectiveness in Response to Omalizumab (PROSPERO) in individuals with moderate-to-severe allergic asthma, 87% of individuals were noted to have a good treatment response to omalizumab irrespective of the baseline FeNO levels. This study’s outcome makes it uncertain regarding the utility of FeNO as a predictor for treatment response to omalizumab [66].

Mepolizumab is a monoclonal antibody targeting IL-5, which has effectively decreased asthma exacerbation rates in severe eosinophil-driven asthma. It is approved for individuals six years and older with severe eosinophilic allergic and non-allergic asthma with or without oral glucocorticoid dependence. Another IL-5 antagonist that is approved for use is reslizumab, which also targets eosinophilic differentiation, proliferation and chemotaxis, and survival. Mepolizumab is a subcutaneous formulation, whereas reslizumab is an intravenous formulation [67,68]. Benralizumab is an IL-5 receptor antagonist which prevents the binding of the IL-5 molecule to its receptor, and it also exhibits antibody-dependent cellular cytotoxicity against basophils and eosinophils. It is approved for individuals with severe asthma 12 years and older [69].

Surprisingly FeNO has not been shown to predict the response to the anti-eosinophil agents confirming that the pathway of the eosinophilic activation and NO induction are different. In the DREAM study, the FeNO readings were unaffected by mepolizumab treatment; hence it cannot even be used as a biomarker for response to treatment. It was also noted that baseline FeNO levels greater than and equal to 75 ppb did not predict response to treatment to anti-IL-5/IL5 R biologics [70,71,72].

Dupilumab is monoclonal antibody targeting the IL-4 receptor alpha, which acts as a common receptor for the IL-4 and IL-13 pathways and hence interferes with IL-4/IL-13 responses. Dupilumab is approved for individuals with allergic and non-allergic eosinophilic asthma, with FeNO of ≥25 ppb, as well as in those who are oral corticosteroid dependent regardless of BEC. It is administered every two weeks subcutaneously [62]. In the LIBERTY QUEST trial, which was a phase 3 study, it was noted that individuals with a baseline FeNO of at least 25 ppb were noted to have approximately 50% reduction in the risk of exacerbation in comparison to placebo whereas individuals with FeNO <25 ppb, there was no significant difference noted. Due to this finding, FeNO can be potentially considered as a biomarker to predict response to dupilumab. It has also been noted that FeNO levels in Dupilumab treated individuals reduce over time from their baseline suggesting that it is also a good pharmacodynamic biomarker of dupilumab therapy [73].

Tezepelumab is a recently approved biologic therapy for severe persistent asthma, which targets TSLP. This molecule targets this epithelial-cell-derived cytokine associated with the multiple downstream pathways linked to asthma pathogenesis. It has been approved for adults and children at least 12 years and older without any phenotype or biomarker limitation. Pretreatment FeNO levels have been shown to predict response to tezepelumab, and like dupilumab, the FeNO levels are reduced in comparison to the placebo group with treatment [74]. Some other investigational anti-IL-13 monoclonal antibodies, like lebrikizumab and tralokinumab, have also completed phase 3 trials in individuals with uncontrolled asthma, however, have not been approved because inconsistent efficacy in the different trials [75,76]. However, a reduction in absolute FeNO values was noted when treated with anti-IL-13 monoclonal antibodies [77]. 

To date, studies have focused mainly on the utility of FeNO in predicting treatment response to biologics with little understanding of FeNO dynamics and interpretation during biologic treatment. In general, higher baseline FeNO values were found to be associated with greater asthma control and reduced risk of exacerbation especially for dupilumab, omalizumab and tezepelumab [78]. FeNO levels remain detectable during treatment with anti-IL5, anti IL4/13 and anti-IL-13 treatment [78]. Similarly, its role in predicting switch of biologic treatment is another area of future research. Considering its cost-effectiveness and ease of use, there is a need for future studies to explore the role of FeNO in long-term follow-up of individuals with asthma on biologic treatment.

## 7. Conclusions

FeNO is a readily available, easy to perform, reproducible and a point of care biomarker. In individuals with asthma, FeNO levels can be used to support asthma diagnosis, predict lung function decline, evaluate risk of poor asthma control and future exacerbations, assess response and adherence to ICS therapy, guide stepdown of the ICS therapy and facilitate choice of certain biologic therapies. As FeNO levels can be affected by a wide variety of confounders and its use as a biomarker may be enhanced in conjunction with clinical findings as well as with other biomarkers of asthma.

## Data Availability

Data sharing not applicable.
